# Being captured by queer kinship: Margaret Lowenfeld and Margaret Mead

**DOI:** 10.1177/09526951251328114

**Published:** 2025-04-10

**Authors:** Katherine A. Hubbard

**Affiliations:** University of Surrey, UK

**Keywords:** kinship, Margaret Lowenfeld, Margaret Mead, Rhoda Métraux, queer history

## Abstract

Margaret Lowenfeld (1890–1973) and Margaret Mead (1901–78) met in 1948. This eventful first meeting in London was the start of a fascinating working friendship, albeit a somewhat uneven one. The two women share particular similarities across their careers, including their positions as women in their respective fields of psychology and anthropology, though Mead was notably more renowned. They also both had substantial and long-lasting relationships with other women. In this article, I draw primarily upon archival resources of interviews with both Mead and Rhoda Métraux conducted about Lowenfeld following her death. In doing so I argue how such material not only reveals the type of relationship between Lowenfeld and Mead, but also raises questions about how lesbian relationships are historically understood. In recognising the queer worlds of these women, it is possible to extend historical thinking about the lesbian relationships they had. Crucially, it also demonstrates what a lesbian feminist historical approach uniquely provides. In addition to this, by likewise recognising myself as a queer feminist, it is possible to reveal the reflexive and emotional queer kinship which extends between historian and subject.

## Introduction

In 1948, Margaret Mead arrived in London for the World Federation of Mental Health Conference. It was at this conference that Mead and Margaret Lowenfeld met for the first time, and it has been described by those writing about Lowenfeld as particularly pivotal (e.g. Lowenfeld, 2013). However, this meeting would not have happened if it had not been for Lowenfeld’s steadfast determination and ability to drive at great speed through Central London. For the conference, Lowenfeld had arranged a display of ‘malaise’ (general illness) case studies of her Mosaic Test (1954) at the Institute of Child Psychology (ICP) and invited Mead to attend the ICP to view them. Mead, however, had no intention of accepting the invite. Though she had seen the title of Lowenfeld’s book *Play in Childhood* (1935), she was not familiar with her work. She therefore sent Edith Carr instead as a ‘surrogate’. Carr was a ‘gifted amateur’ who had read *Play in Childhood* and had begun to research childhood autobiographies. Nevertheless, Lowenfeld was not impressed. Mead reported that Lowenfeld took ‘one look at Edith’ and said, ‘I’m going to get Margaret Mead’:

And she literally captured me in her car and drove hell for leather through London … ferocious, a good driver, but ferocious. She literally kidnapped me. I was busy doing something else and here is this – woah and she grabbed me and took me off. And I saw most what the mosaics could do.^1^

So was the start of a fascinating working friendship. One where Mead recognised the value in what Lowenfeld was producing at the ICP, yet simultaneously had to fend off Lowenfeld’s attempts to ‘capture’ her, both literally as demonstrated in the above description, but also in a professional capacity. In this article, I use this concept of being captured to untangle some of the complexities of lesbian feminist historical relationships, that is between Lowenfeld and Mead, plus several other women important to this story.

Lowenfeld first met Mead in London, and the event shaped their working lives. It was a memorable meeting that led to a professional, and sometimes collaborative, friendship. It also, I argue, produced a sort of kinship between queer women working in difficult circumstances. I similarly ‘met’ Lowenfeld in London. It has led to a collaboration of sorts and shaped my working life in various ways. It has, I feel, also led to a sort of queer kinship across a historical divide. I cannot claim to have known her (or to have been kidnapped by her), but I nonetheless would argue Lowenfeld has ‘captured’ me in ways that Mead also reported having to resist. Mead has had similar effects on queer scholars. In *Margaret Mead Made Me Gay*, Newton (2000: 3) ‘records the intellectual journey into which I was seduced, not by Margaret Mead in person, but figuratively by her, in the very lovely sense of the word figuratively’.^2^

This article therefore aims to provide nuanced and anti-heteronormative analysis of the queer network and friendship between Margaret Lowenfeld and Margaret Mead. I focus primarily on Lowenfeld here as the central figure but crucially draw upon the relationships with Mead, Ville Anderson, and Rhoda Métraux in order to untangle this small network of women active in the mid 20th century. In doing so, I discuss the queer intricacies and historical challenges of such an undertaking. Crucially, I reflect on how it is only through my own lens as a queer feminist woman that such a narrative can be revealed and consider the absolute necessity of *lesbian* as an analytic tool – something that I demonstrate was not possible in the 1970s.

## Emotionality in/and/as archives

Emotive responses in relation to figures of the past have been articulated by other historians and scholars. Lepore (2001: 133), for example, questioned whether those conducting biography or microhistory (that is, those with a more specific, niche, or individual-level interest) can love too much. She argued that biographers in particular ‘are notorious for falling in and out of love with the people they write about’. But emotionality is perhaps especially vital for queer history, as, to quote Cvetkovich (2003: 242), ‘understanding gay and lesbian archives as archives of emotion and trauma helps to explain some of their idiosyncrasies or, one might say, their “queerness”’.

Dinshaw (1999) has articulated an affective, and perhaps an affectionate, ‘touch across time’ for historians, one that draws upon a queer historical impulse. Drawing upon such work, Love (2009: 12) paid closer attention to 20th-century texts and argued in *Feeling Backwards* that when we think about the emotionality of the queer archive, ‘personal encounters and the feelings they elicit stand in for theories of history and of the social’. The queer impulse resonates with queer historians in particular:

The longing for community across time is a crucial feature of queer historical experience, one produced by the historical isolation of individual queers as well as by the damaged quality of the historical archive. (Dinshaw, 1999: 37)

Likewise, Love (2009: 37) argues that Dinshaw presents figures of the past as ‘yielding to, or even warming to, the touch of the queer historian’. In this article, I feel especially drawn to such a presentation of queer figures of the past. It is true that here I present a historical analysis that ‘touches the past’, but I also wish to give some agency to the people I discuss. Just as Lowenfeld captured Mead, so too have I been captured. There is undoubtedly a queer impulse at play here, a queer instinct or, as Koaureas (2014) argues, a reading between the straight lines of the archive. I also argue that in the development of such deeply connected personal historical work, a type of queer kinship is formed between the researcher and the subject. Perhaps Lepore (2001) is right, and historians can love too much. Or perhaps, for queer scholars, it isn’t love, but rather queer kinship complicated by love, community, and desire.

Recent work by Jenn Shapland in *My Autobiography of Carson McCullers* (2021) beautifully untangles the complex relationships of conducting historical work on queer figures that feel close in many ways, but are nevertheless separated by time, existence, and direct communication. Akin to the queer impulse and touches across time, Shapland’s work uniquely presents her own mental health, well-being, sexuality, sense of discovery, and exploration into the very fabric of the (auto)biography of McCullers. As a reclamation of lesbian figures, histories, and complex narratives, Shapland places herself into the narrative in ways that recognise the emotionality of queer history.

Such recognition of our own positionality and the contexts in scholarly work is produced is, in my view, essential – it is a detriment to scholarly work that we strip the writer from the written. To embrace the value of subjectivity and the importance of intersubjectivity, plus the emotive basis for why particular topics are sought out, is only to enhance historical projects. The analysis I present here is the product of (a) a Wellcome grant, albeit one restricted to archives in the UK; (b) an incredible amount of persistence in a context blighted by a pandemic; and (c) a queer kindship that I could not forgive myself for giving up on.

Thinking historically about figures of the past is complex and, understandably, some figures attract more historical attention than others. Mead shot to fame in the late 1920s with the publication of *Coming of Age in Samoa* (1928) and *Growing Up in New Guinea* (1930) as one of the early proponents of cultural relativism. She became internationally known and was perhaps the most famous anthropologist in her own lifetime, and her celebrity continued throughout her career. Indeed, Mandler (2013) describes her as ‘certainly one of the most famous women in the world’ in the 1960s. There has been a wealth of work conducted on the life and work of Mead, quite understandably given her remarkable life and contributions to anthropology. These include her own accounts, for example *Blackberry Winter* (1972 and 1974a). There are also other book-length biographies (Banner, 2010; Bowman-Kruhm, 2003; Howard, 1984), as well as the memoir of Mead and her third husband, Gregory Bateson, written by their daughter Mary Catherine Bateson (1984). In addition to these, there have also been books published specifically on Mead and Ruth Benedict’s relationship (Banner, 2010; Lapsley, 1999), the letters of Mead (Caffrey and Francis, 2006), Mead’s Episcopalian faith (Coffman, 2021), Mead’s research and relationships during the Second World War and Cold War (Mandler, 2013), posthumous controversies around her anthropological arguments (Shakman, 2009), and her position within Boas’ group of ‘unconventional women’ students and ‘renegade anthropologists’ (King, 2019).^3^

Similarly, there have been publications memorialising Lowenfeld, but to a much lesser extent (e.g. Traill and Hood-Williams, 1973; Urwin, 2004). There have been efforts to re/publish her written work (Lowenfeld, 1979) and place the ICP within histories of psychology more concretely (Lowenfeld, 2013). Attention has been paid to the psychological tests she developed and their legacies (Bowyer, 1970; Hutton, 2004), and I have been particularly interested in her role within projective testing and queer histories, and as a woman in psychology (Hubbard, 2017, 2018, 2020).^4^ Yet Lowenfeld has attracted rather less attention from biographers. In fact, there have been two attempts to write a biography of Lowenfeld, one by Gerald Sanctuary in the 1970s following Lowenfeld’s death and another by Barbara Evans a few years later. However, both were initially set up as dual biographies covering both Lowenfeld and her sister Helena Wright. Both attempts were left incomplete and never resulted in publication, though Evans did publish a fascinating biography of Wright (Evans, 1984).

The central focus in terms of content for this article is the so called ‘Sanctuary Tapes’, the recorded interviews conducted by Gerald Sanctuary in 1967 and 1977 when he was undertaking his never-to-be-completed dual biography. I pay particular attention to the interviews with Mead and Métraux.^5^ Not all of these tapes survive, and it appears from the archive notes that some were given to Evans for the subsequent biography attempt.^6^ What the archive is able to offer for this article is not a true representation of the working friendship between Lowenfeld and Mead. The interviews themselves, however, offer a relatively rare historical opportunity. They are substantially more informal and allow greater insight into Mead’s thoughts and perceptions of Lowenfeld, beyond the formalities of the obituary she wrote for Lowenfeld (Mead, 1974a). They are of course still going to be crafted, constructed, and created in the particular context of the interview, and there is no ‘true’ or ‘real’ version of Mead’s beliefs that can exist. It is also important to also be mindful that this is a distinctly one-way exchange. Lowenfeld has had no such opportunity to discuss Mead posthumously.

In capturing these women within the purview of queer history and recognising their lesbian relationships, it’s possible to also see what is at stake without such an affirmative queer historical undertaking. There is of course a need to consider very carefully the risks of presentism and the attribution of sexual identities onto figures of the past. Despite significant relationships with women, neither Lowenfeld nor Mead ever outwardly identified themselves with an explicit sexual identity, though this is not surprising.^7^ While the concept of ‘homosexuality’ had very much emerged by this point, very few would have identified themselves with such terminology, especially in an affirmative and positive manner (Waters, 2012).^8^ Dinshaw (1999: 1) argues the queer impulse is all about making connections and touching across time ‘between, on the one hand, lives, texts and other cultural phenomenon [*sic*] left out of sexual categories back then and, on the other, those left out of current sexual categories now’. The temporal space between when sexual categories came into existence and when open affirmative identification with such categories became widespread is therefore ripe for consideration despite the associated challenges. In many ways, these women resisted categorisation; Lowenfeld struggled to articulate a definable identity in both her personal and her professional life. It is therefore not my intention to apply an identity to her in a way that implies my historical hindsight ‘knows’ her better than she knew herself. Yet I do wish to demonstrate what a queer feminist analytical perspective can reveal when we are less afraid of applying some terms in order to frame historical analysis. In using a queer affirming perspective, I provide an antithesis to common heteronormative narratives. I therefore use *queer* and *lesbian relationship*, with deep consideration of the contexts in which these relationships occurred and of the issues of applications of potential identities onto figures of the past while actively retaining an anti-heteronormative perspective.^9^

## Introducing Lowenfeld

Margaret Lowenfeld, or ‘Madge’ to her family, was born on 4 February 1890 in Lowndes Square, London. She is often remembered as having a ‘difficult’ childhood having experienced various illnesses. Her parents divorced in 1902, which led to some challenging relations, and she was described as experiencing ‘deprivations of the rich’ (Hubbard, 2020: 69). Like her sister Helena Wright, she attended Cheltenham Ladies’ College, and the two of them were highly resistant to their father’s attempts to prevent them going to medical school and living independent lives (Evans, 1984; Lowenfeld, 2013; Urwin, 2004). In 1912, Lowenfeld entered the London School of Medicine for Women. Shortly after graduating in 1919, she was called to manage the staff at the family home in Poland following the end of the First World War (see Lowenfeld, 2013).^10^ Her experiences of Poland and the poverty she witnessed, including the number of orphaned children, inspired her to forge her career in child psychotherapy. On returning to London in 1921, she experienced a mental ‘collapse’ similar to those she had had as a teenager (Urwin, 2004). In 1928, she opened her Children’s Clinic for the Treatment and Study of Nervous and Difficult Children, which later became known as the Institute for Child Psychology, in London, and which worked mainly with children from poor backgrounds (Lowenfeld, 2013; Traill and Hood-Williams; 1973). The ICP ran as a child guidance clinic that trained child psychotherapists in Lowenfeld’s techniques, tests, and approaches to child psychotherapy.

In the 1920s and 1930s, Lowenfeld was also a member of the British Society for the Study of Sex Psychology, a progressive group characterised by a ‘refusal to accept conventional dogma about sex and gender’ (Hall, 1995: 666).^11^ During this time, rivalries began between the different schools of thought in psychology, and although Lowenfeld was to meet Carl Jung and Alfred Adler in the 1940s, she did not consider herself psychoanalytic and did not align herself with any particular school. Nonetheless, she contributed two relatively well-known tests – the Lowenfeld World Technique (1979) and the Mosaic Test (1954) – plus kaleidoblocs, poleidoblocs, and thinking about the protosystem (see Hutton, 2004; Lowenfeld, 1954, 1979). In many ways Lowenfeld was a pioneer in play therapy, especially with regard to sand-play techniques. She centralised child-led play and the ICP was unique in its approach, including, for example, a wet room where children were free to play with water. Her collection of toys for the Lowenfeld World Technique and the Mosaic Test were believed to be scientific and, in some ways, culture-free instruments – a belief strengthened by her correspondence with Mead (Joice, 2023). Her work was especially influential in the decades following the Second World War, as concerns around children and childhood increased in Britain at this time. Joice (2023) frames Lowenfeld during this period within attempts to use visual imagination to support the children’s mental health. Indeed, as Joice articulates, Lowenfeld fundamentally viewed play as not only therapeutic, especially in response to trauma, but also preventative, and argued play ought to be integrated more closely into educational and psychiatric services.

However, Lowenfeld did not disseminate her ideas with much force (Evans, 1984), and was not recognised as a major figure (Bowyer, 1970; Hutton, 2004), despite her techniques often having a global influence (Joice, 2023). By 1970, towards the end of her life, Lowenfeld suffered from confusion and alteration of mood (Evans, 1984). She was moved to a nursing home near her sister’s house in 1972 and died a few months later on 2 February 1973.^12^ The ICP was disbanded only six years later, having received a lack of support from the psychological community, and endured tensions in its organisation and management. In 1989, a documentary was filmed about her work and her influence upon the field of child psychotherapy by the Margaret Lowenfeld Trust (who are still running today).^13^

Despite a relative lack of recognition within the psy-disciplines, Lowenfeld’s tests were applied in anthropological fieldwork and Mead was instrumental in this, having been given a demonstration of the Mosaic Test during their first unforgettable meeting. Influenced by Mead, Rhoda Métraux, who is another important figure in this focal history, used the Lowenfeld Mosaic Test during her fieldwork in Tambunum, Papua New Guinea (see Lemov, 2011 for a history of projective methods in anthropology). Mead officially remembered Lowenfeld in the obituary published in the *Journal of Clinical Child Psychology*. Here Lowenfeld was described as innovative and imaginative, yet she continually experienced a ‘struggle between the unstinting attention she gave her patients, her urge to expand her work in every possible direction, an imagination that far exceeded her bodily strength, a shortage of funds and a terrific sense that time was running out for the western world, if not the entire world’ (Mead, 1974a: 57). Reflecting on their first meeting in 1948, Mead avoided the exact circumstances but recognised that ‘Margaret Lowenfeld created a whirlwind intellectual atmosphere’ (ibid.).

The same year Lowenfeld met Mead, she also met another woman who was to have a substantial impact on her life: Ville Anderson (see Lowenfeld, 2013). Having met Lowenfeld in Denmark (Anderson was Danish), Anderson moved to London to train at the ICP in the 1950s, and upon completion she stayed at the ICP ‘as a psychotherapist, research worker and assistant to Margaret Lowenfeld, remaining with her as a colleague, living companion and confidante until the time of Margaret Lowenfeld’s death’ (Lowenfeld, 2013: 14). Such descriptions of their relationship are common. Anderson is often referred to publicly as a close friend, colleague, or carer of some kind, due to Lowenfeld’s apparent fragility. For example, Urwin (2004) states Lowenfeld ‘was supported by her close friend and living companion, Ville Andersen, a Danish citizen who had trained at the ICP in the 1950s’. In previous work, I have deliberately articulated their relationship as more than mere friend or colleague and conceptualised them romantically (Hubbard, 2017, 2020). Their relationship spanned decades and was notably intimate. The two of them lived together and split their time between their Harley Street flat and ‘Cherry Orchards’, a house in Cholesbury, Buckinghamshire, which Lowenfeld had bought when her mother died. It was in this house that Anderson cared for Lowenfeld in the final years of her life. Lowenfeld’s death is believed to have been particularly hard for Anderson. After her death Anderson started to archive Lowenfeld’s papers, letters, and work, including childhood letters that Lowenfeld had given her. In explicitly naming this relationship as romantic and lesbian, I continue to expand the potential for analysis and position Lowenfeld within queer history.

Mead’s significant relationships with women became especially relevant upon Mead’s daughter’s discovery of her relationships with other women following her death (Bateson, 1984; Grossman, 1985). This included Mead’s relationship with her mentor and fellow anthropologist Ruth Benedict, which has been recognised in literature about her (e.g. Banner, 2010; Lapsley, 1999), as of course have her three marriages, most significantly to Gregory Bateson, with whom she had her daughter (see Bateson, 1984). It is worth noting that Mead and Benedict remained lovers into her marriage to Bateson (ibid.), and their broad attitudes to sex have led some to describe Mead and Benedict as ‘sexual iconoclasts’ (Newton, 2000: 8). Some scholars have considered Mead’s relationship with Benedict in particular (Lapsley, 1999), and Mead has broadly been considered bisexual, or, as she called it, of ‘mixed type’ (see Banner, 2003, 2010; Coffman, 2021; Lapsley, 1999; also Grossman, 1985). Mead has been framed as a distinct queer figure, and her relationships with other women have been described across research and biographies about her (Banner, 2010; Lapsley, 1999; see also Banner, 2003 for an in-depth consideration of Mead’s views on sexuality). Here, I continue to extend my queer reading to Lowenfeld but also consider the queer intersubjectivity between her and Mead.

## ‘Principal theoretical supports’: Margaret Mead and Rhoda Métraux

The relationship between Lowenfeld and Mead was, perhaps understandably, a rather uneven one. Mead at the point of their meeting was internationally recognised and had conducted some of her most famous work decades before meeting Lowenfeld. As highlighted, Lowenfeld’s career had not had the same level of success, despite her potential:^14^

She had all the making of a school founder. Without the ability to found a school.…

Anna Freud and Melanie Klein had the backing of the psychoanalytic institute. The establishment. They even stayed together when their theories were mortal enemies. But she [Lowenfeld] never had anything like, she was, she never was academic, she never had the discipline. A medical education in England is not an academic education. You don’t really learn much about research or academic cooperation or anything.

It is apparent in the interviews that Mead was conscious of how Lowenfeld at times may have attempted to capitalise on Mead’s success and academic know-how, and somewhat ‘ride her coat tails’. Mead certainly supported Lowenfeld’s grant applications, helped coordinate three visits to the US, stayed with Lowenfeld at Cherry Orchards, and promoted her tests among anthropologists. She described their relationship thus:

I was one of her principal theoretical supports, for 25 years. And I was one of her principle [*sic*] practical supports in terms of getting her funds, and trips over here and things of that sort. But in a sense, we were hardly friends, in the sense of any degree [of] intimacy. But when you work with this kind of material, you know it’s a kind of substitute for intimacy. You’re exchanging so much emotional content to somebody. Talking about a case, it’s a kind of intimacy.

When asked about being captured or appropriated by Lowenfeld, Mead highlighted that her first literal kidnapping had been ‘very worthwhile, I saw something that stayed with me forever’. The ICP at the time was struggling financially, and within months Mead had organised £5000 to be sent to support the work there and was corresponding with Lowenfeld about a US visit. Yet frustrations were clearly evident:

Oh I used to get furious with her.…

I would get tired of being given lectures about stuff we’d all known for years. You know, I’d get bored by it. But I also got tired of her fighting with psychoanalysis instead of facing the fact she was doing something different.… It was a waste of energy, a tiresome waste of energy. So periodically we would get into an argument.…

She as always trying to appropriate me. She’d go out into the United States and meet someone I had never met, or heard of, and decide they were wonderful people and propose that I write a book with them. Maddening! And I would say, look, I’m not going, I don’t know anything about this person, I’m tired of your enthusiasms, 50% of which are good and 50% of which are bad and I’m not going to be taken around the country being proposed as a co-author, co-project.…

Well you know, in a sense she wasn’t quarrelsome, it wasn’t a major issue. And you could stamp your foot and object to things.

In a letter to Mead’s daughter in 1957, Mead said, ‘And in this case there is also Margaret Lowenfeld to cope with who wants me to do things in Denmark’ (Caffrey and Francis, 2006: 361), further indicating some frustration with Lowenfeld. Nonetheless, despite Lowenfeld’s efforts to capitalise on such a working relationship, the frustrations, and the annoyances, Mead maintained their professional relationship and described Lowenfeld as a ‘professional friend’.

I didn’t love Margaret. I valued her. Enormously. I had a great deal of admiration for her and I thought she [was] terribly valuable and she developed something very important and new and she had to be helped at every turn but I didn’t have much of an impulse to talk to her about my life. There wasn’t time. And because I didn’t talk to her about my life she didn’t in a sense talk to me about *her* life. She talked to me about her life in a sense of theoretical points. And she told me stories about her father occasionally. And they came into a conversation like a case might come in, or a trip to somewhere might have come in, you see. They weren’t long, there are a few letters which are reasonably personal but they are from when she was had a lot of problems on her hands, she might mention them. But we were definitely, professional friends. Without time to be anything else. If I lived next door to her then there might have been more time to talk about all sorts of things. But we desperately always pressed.

At times when Mead was not available, Lowenfeld would instead engage with Rhoda Métraux, who, Mead hypothesised, resented this slightly. Mead identified Lowenfeld here in the conversation as ‘a bit of a snob – in the best sense of the word, in order to get to the top’.

Upon reflecting on what Lowenfeld was like as a person, Mead and Métraux produced a range of contrasting and sometimes conflicting descriptions. Métraux met Lowenfeld two years after Mead, in 1950. Métraux’s impressions of Lowenfeld echoed much of what Mead described in terms of being ‘appropriated’. Yet she was somewhat more defensive about Lowenfeld’s intentions. She jovially explained Lowenfeld ‘would take up 36 hours of your time in a day’. Yet despite this, and the growing sense in the transcript that Lowenfeld was being painted as a manipulative figure, she was very clear she did not view this as exploitative:

In a sense that if she saw there was something available in a person or situation that met her need, which is very different from exploiting a person. We usually say when someone uses a person that’s exploitative, I don’t think its exploitative.

A key contradiction in how Lowenfeld was framed concerned concepts of strength and perseverance. Lowenfeld was described as being doggedly determined and persistent, but also weak and frail:

Métraux: ‘There was a discrepancy between her enormous appetite and her wanting to take over a scene and her strength. And then by 8, 9 or 10 at night she was done. Like that!’

Mead: ‘She would collapse suddenly.’

Such concepts also appear to have been drawn into juxtapositions between Lowenfeld’s femininity and her somewhat masculine traits. For example, Gerald Sanctuary said he spoke to someone who reported Lowenfeld ‘had a very feminine personality and knew very well how to relate to men’. In response, Mead replied, ‘There’s no doubt about that’, and Métraux confirmed, ‘She was very feminine.’ Mead elaborated,

She was very feminine. She was *a very strong woman*. She wasn’t mannish, you know [original emphasis].

Later in the conversation, Mead also reported that Lowenfeld ‘didn’t have a male identification but identification with her father’s vigour, imagination and entrepreneurship’. But when Mead restated, ‘She was strong’, Métraux rejected the idea immediately:

She was not strong. She was frail. All this all this, all the strength was psychic energy that carried her.… She was *not* physically strong [original emphasis].

When thinking about her physicality, Mead corrected herself in thinking that Lowenfeld was particularly tall:

Margaret was very, now I think probably one experienced her as bigger than she was. She was so expansive and so forth. But still, she was much taller than I am.

The contrast therefore appears to lie with an emotional and intellectual energy that was unmatched by her outward feminine persona and physical weakness. She was described as being dogged by illness her whole life, and certainly there is archival evidence of her experiencing mental illness and seeking psychotherapeutic support (Hubbard, 2020). Métraux summed up, ‘What is strong with her is *will*, and the light and the passion and concerns, and entering into people’s lives, but as for physical strength’ (original emphasis).

The sense that Lowenfeld had a particular strength in gaining access to people’s internal lives was especially highlighted and valued by both Mead and Métraux. This was unsurprisingly emphasised with particular regard to children. Métraux’s first impression of her was as follows:

Well you know her speed of perception was one thing and the ease at which you could enter into a conversation about anything of interest, to you, to herself, about the world about anything. Which she would then absorb and then elaborate on in all kinds of ways.…

She was extraordinary in how she captured a child in a short period of time. Children would easily express what was in their heart as well as in their mind to her. She didn’t have to take over – she entered in.

In rounding up their impressions of Lowenfeld as a person, both Mead and Métraux once again drew upon the concept of capture, and Mead concluded,

She was captivating, that is the correct adjective. Because she had great charm, just plan ordinary charm.

Then, in reference to Mead being kidnapped by Lowenfeld, Mead reflected,

That wasn’t charm that was muscular, but she did have charm and she had a great deal of charm and style. But she was captivating at every level.… Her ideas were captivating. I think that’s a good word.

The picture painted here is therefore one of a rather complex character. Mead and Métraux commented on the captivating energy of Lowenfeld as well as her insightfulness and giftedness with children, which appear to have gone hand in hand. Lowenfeld’s wilfulness was also highlighted, indicating an overall singular character who is difficult to place or easily define.

Lowenfeld is at once both muscular and charming, frail and feminine. It is also notable how her femininity was emphasised, and both Mead and Métraux were keen to reiterate that she was not ‘mannish’. In fact, Mead had a complex understanding of ‘mannishness’, as she struggled with conceptualising how lesbians were not necessarily ‘mannish’ by virtue of their sexualities (Banner, 2003). This goes some way to explaining her defensiveness of Lowenfeld’s femininity, as she herself was also very feminine and appears to have had difficulty of configuring lesbian attraction and masculinity and femininity (ibid.).

## ‘An impossible Danish spinster’: Ville Anderson

In previous work I have articulated the relationship between Lowenfeld and Ville Anderson as distinctly queer and romantic. The pair first met in 1948, and, as Mead related, from 1952 onwards, ‘the story of Ville’s life was Margaret’. The two were exceptionally close, and Ville cared for Margaret and her work substantially throughout the rest of her life (Hubbard, 2020). Following Lowenfeld’s death, Anderson moved back to Denmark to the thatched cottage that she and Lowenfeld had often holidayed in together. In the interviews it is clearly articulated the extent to which the publications of Lowenfeld’s tests and theoretical contributions were down to the work of Anderson, and this was echoed in her official obituary (Mead, 1974b):

Ville did it. Ville got the poleidoblocs *made*, she had the mosaics *made* [original emphasis].…

She has a real sense of precision on and materials and elegance and execution.

Mead framed Anderson and Lowenfeld’s working relationship as one in which Lowenfeld would imagine and theorise, and Anderson would execute and enact. This could easily be framed as another uneven professional relationship, but Mead conceptualised this somewhat differently to her own relationship with Lowenfeld, as she strongly recognised the commitment and devotion Anderson had towards Lowenfeld.

Margaret she looked to people as if she had an enormous ego, I think, sometimes, but what she had was an enormous dream.

And while Lowenfeld was committed to this dream, Ville was committed to Margaret.

After all, Ville learned something about Margaret’s work by some minor accident I think, and then went and sought out Margaret and had the, capacity to say this is what I want and this is what I want to work on all my life.…

I think what she [Anderson] wants is for people to see she has contributed through her devotion. She has a sense of inferiority of her own capacity but not of what she did.

Anderson also clearly has a high level of respect and reverence for Mead. In 1975, two years after Lowenfeld’s death, Anderson wrote to Mead thanking her for her support. Mead reported that it read,

Our mutual friend Margaret once said to me when I felt the responsibility for carrying out her wishes after she had gone rather heavy ‘you can always reply on Margaret Mead, she can tell you when you are wrong and support you when you are right’. And that is what you have done. I don’t know if I could have survived this struggle without you. Have often thought about running away from it all, but that would mean to let Margaret and her lifelong work down so I cannot.

Anderson was indeed supported by Mead following Lowenfeld’s death in maintaining her ‘executive position’ as the ‘final authority’ on all things Lowenfeld. There appears to have been some difficulty in organising the Lowenfeld Trust and the ICP. Such personal tensions were not new, and during Lowenfeld’s lifetime there appear to have been personality clashes and power struggles at the ICP.

Sanctuary, however, appeared to be somewhat ignorant of the deep emotional bond between Lowenfeld and Anderson. In the interviews with Mead and Métraux, he reports he had a disastrous meeting with Anderson in Denmark because he had to leave suddenly upon the news that his father-in-law had been taken seriously ill. Anderson was distraught and had ‘tears pouring down her face’. She was resistant and protective, clearly uncertain and defensive – in Mead’s words, ‘hostile and jealous’. Anderson guarded much of the key archival materials that Sanctuary wanted for the project, and he reported making copies of documents without her permission. Both Mead and Métraux provided instructions and advice on how to persuade Anderson to continue with the interviews and help Sanctuary write the book. They all recognised that Anderson was key to being able to write fully and comprehensively about Lowenfeld. Anderson was evidently distraught, and they all understood that she would fully withdraw any cooperation if it became known Sanctuary had obtained materials without authorisation. Mead advised him to not share any of his written work with her directly. Anderson was evidently deeply bereaved, yet few sympathies appear to have been extended to her. Sanctuary framed her and their relationship thus:

Ville doesn’t immediately have an attractive personality. It’s easy to side with the people who don’t like her personality and ‘perceive the paranoid tendencies’ in her and dismiss her as an impossible Danish spinster. That isn’t fair to her, I’m quite sure she contributed an enormous amount to Margaret Lowenfeld, for being there as a chopping block, for being there as a support, for being there as an organiser, a companion.

He also theorised that of course Lowenfeld would have ‘run out of steam’ earlier than she did without Anderson. In response Mead somewhat curtly stated, ‘Ville was indispensable’.

As archival researchers, it can certainly be frustrating when materials are not available, or obtuse obstructions seem to get in the way. But that is the very nature of archives. This is especially true for those with queer contents and themes. Sanctuary exclaimed that Anderson’s extracts were ‘appalling examples of censorship’. However, I would counter that. From the archival materials I have been able to access, I have still been able to glean extensive insight into the life, work, and loves of Lowenfeld. What has been lacking before now is queer feminist insight and the recognition of the relationship Anderson had with Lowenfeld. It is precisely through viewing this relationship as lesbian, romantic, and intimate that Anderson’s actions and emotions may be more clearly understood – as those not of an ‘impossible spinster’ but of a bereaved life partner. The following sections draws particular attention to this, as I argue reflexive queer feminist analysis has been crucial in contextualising and understanding the dynamics of the relationships of these women.

## ‘A frequent visitor’: Robin Collingwood

One of the accusations levelled at Anderson regarding the archival material was that she had removed almost everything related to personal relationships. The interviews conducted by Sanctuary were therefore invaluable, as Mead and Métraux elaborated and expanded on more personal details. For example, Mead outlined, and Métraux agreed, that despite Lowenfeld’s ‘gargantuan interest … in a tremendous number of children’, they never got the sense Lowenfeld missed having children herself. Lowenfeld’s personal life, including her romantic and sexual relationships, therefore came under discussion.

One of the relationships that they discuss is the one Lowenfeld had with the philosopher Robin Collingwood (1889–1943). The two met in 1917 in the Student Christian Movement, but by 1918 relations had become ‘acrimonious’, according to Hayward (2020), and, probably not uncoincidentally, Collingwood was engaged to and married Ethel Graham that same year. Their relationship has been framed in romantic and sexual ways. For example, Inglis (2009) distinctly frames their relationship as an affair, citing gossip around the pair. Hayward (2020), while recognising their relationship may initially have had a romantic and/or sexual nature, instead frames it as a friendship. Specifically, he argues that they had a great deal of shared interest in understanding emotional life, and Lowenfeld’s work at the ICP aided them both in thinking complex ideas of feeling and thought. In *Play in Childhood* (1935) Lowenfeld used Collingwood’s concepts, and Collingwood even assisted in the original establishment of the ICP in 1928. He was a regular visitor, and his wife was at one point on the board of governors (Hayward, 2020). In Lowenfeld’s obituary, Mead identified Collingwood as a ‘frequent visitor and critic’ of the early ICP (1974a: 56).

Mead and Métraux promised to assist with managing Anderson in attempt to ensure access to the materials they believed were relevant to this period. However, Mead said that Anderson had already destroyed letters from Collingwood to Lowenfeld: ‘They were “marked for destruction”' and it had been done before Mead arrived (presumably in Denmark to assist Anderson). Métraux first introduced their relationship as one of intellectual collaboration, but both Mead and Métraux were keen to dissuade Sanctuary from the idea that Lowenfeld was passive in such an intellectual exchange.

Métraux: ‘One of the first things I discovered about her was her relationship, something about her relationship with Collingwood. Now Collingwood had been a great discovery for me.’

Mead: ‘I think Collingwood was one of the most important intellectual influences in her life.’

Métraux: ‘And she was one of the most important intellectual influences in his life. When reading *Principles in Art* [published 1938].’

Sanctuary: ‘I perceive that he was a major influence on her. I didn’t realise she influenced him.’

Mead: ‘It would be quite impossible for her to be influenced by someone and not have an influence. It would have been quite intolerable, nobody would have been able to try and influence Margaret without –’

Indeed, Collingwood briefly cited Lowenfeld’s *Play in Childhood* (1935) in *Principles of Art* (1938: 80), highlighting how she had ‘devised a method for exploring the unknown world of children, and has made strange discoveries about the relations of his play to the child’s health’ (see Hayward, 2020). Hayward highlights the perhaps surprising involvement he had with the ICP given his distaste for psychology (ibid.).^15^ Shortly after this exchange, Sanctuary sought some reassurance or advice about the extent to which the intended book should include Lowenfeld’s ‘personal relations, passions and intimate life’, or how far it should avoid the topic. Mead, in characteristically direct fashion, responded, ‘Well she certainly had lovers’, and Métraux concurred, ‘No doubt about it.’ This did not respond directly to Sanctuary’s query, but he appears to have taken it as a direct invitation to further discuss Lowenfeld’s romantic and sexual life, which once again drew attention to Collingwood. Mead continued,

And I think that was something she wanted people to know, those people that knew her well, I remember her saying to me ‘I know a great deal about sex! Every kind of sex!’ She was talking about something else but it was put in the middle of things.… And for a long time, I had an image in my mind, without doing much with it, that Margaret had had a long-standing love affair with someone of great political importance. But I had no notion of Collingwood as being an important part of her life until after she died and I read the accounts. Did you [to Métraux] know how well she knew Collingwood?

A brief disagreement then occurred between Mead and Métraux regarding when Mead first found out about Collingwood, before Métraux concluded this discussion with a summing-up:

I think they became very collegial friends over time. I think she fell, I think she fell, I think since she almost had a … crush, no that’s too girlish. But she fell head over heels in love with him, you know to a wide extent he responded then when he withdrew, he withdrew at some point, he must have mastered it, or seen it through in some way. I have a feeling that they became intellectually extraordinarily related to one another and that part, I knew more about from the beginning of my knowing her.

Mead on the other hand, maintained that she had a sense of a serious relationship that was never clarified or named.

The conversation then moved to another point of departure, whereby a clear difference of interpretation is evident between Sanctuary and Mead and Métraux. Sanctuary explains that ‘you would have expected a person like that to marry. At least I would have expected someone like that to marry.’ Mead and Métraux clearly disagreed with him. Mead responded,

Who could she have married?… Well you know there wasn’t just Englishman around who would marry someone with that degree of passion, of intensity and goal-directedness and everything.

Indeed, while Lowenfeld never married, she did of course spend over two decades with Anderson. It is also interesting that Mead always had the sense of Lowenfeld being in a long-standing and almost secret relationship. Lowenfeld’s defended femininity and Anderson’s apparent masculinity can be cast in a different light here, as they potentially echo femme butch dynamics, which would have been understood as distinctly queer in the early 1970s. To me, as a queer feminist researcher, the dynamics of these relationships and the women involved reads as distinctly queer; and I argue it is evident that some form of queer kinship had developed between Lowenfeld, Mead, and Métraux.

## Lesbian interpretations of heterosexual daydreams

The queer potential and reading across Lowenfeld’s life is something I have argued is ripe for nuanced queer feminist historical understanding (see Hubbard, 2017, 2018, 2020). Even Sanctuary appears to have noticed it, and asked directly,

Do you think the relationship with Ville was a homosexual one?

The moment I first heard this question uttered, sitting in the Rare Materials Room at the Wellcome Library, listening to these tapes, felt like a dream come true. I’d been asking myself this very question. Métraux responded ‘no’ and Sanctuary concurred, ‘No, I don’t think so either.’ Mead’s response, however, was somewhat more revealing:

But with that generation its [*sic*] extraordinary hard to tell. I had some friends who I assume were, had a full-blown relationship and then after they separated years later the woman who was very masculine and very sophisticated and psychoanalyst and some young girl fell in love with her, she was shocked to the depths of her soul as if she had come out of a nunnery.… So you can’t tell but I would doubt it. I think they both lived in these heterosexual day dreams. They weren’t heterosexual realities.

The discussion ends briefly with Sanctuary suggesting that there was probably ‘a degree of attraction that was never overt. Having met Ville Anderson …’, and Mead similarly hedging her bets: ‘I would think so. I doubt there was even a great deal of expression of affection.’

There was therefore certainly some recognition of the queerness of the relationship between Lowenfeld and Anderson – it may not have been overt or explicit, but Anderson herself is at least being framed as having ‘a degree of attraction’ to Lowenfeld. I argue that their spending over 20 years together suggests this attraction went both ways. There has been a relative comfort in associating Lowenfeld romantically with Collingwood in comparison to the relative discomfort of associating her romantically with Anderson, in the literature and beyond, and here I aim to challenge such heteronormativity.

There is one final piece of information that is crucial in understanding this exchange – something that was probably not known to Sanctuary as he asked this question. When asking whether Lowenfeld and Anderson were in a ‘homosexual’ relationship, he was missing one vital point: he was asking two women – *who were themselves in a romantic relationship* at the time. Indeed, in the interview, it is possible, and I argue likely, that it was Sanctuary living in a ‘heterosexual day dream’ – it was not a ‘heterosexual reality’, to use Mead’s own words.

Mead and Métraux first met when Métraux became Mead’s research assistant in 1941, which was ‘the beginning of a long professional and personal relationship that continued until Mead’s death in 1978’ (Mitchell, 2005: 178). They had lived together since 1955, when Métraux purchased a house in Greenwich Village, New York. Mitchell (2005) clarifies that at this time Métraux occupied the upper two floors with her son Daniel and Mead the lower two floors with her daughter Catherine. Later, however, Mead purchased them an apartment to share (Lapsley, 1999). They wrote together, including academic publications (e.g. Mead and Métraux, 1957, 2000) and more public-facing work, most famously the regular articles for *Redbook* from 1963 onwards. It was Métraux who wrote Mead’s biographical sketch in *American Anthropologist* following her death, in which she described the last conversations they had just before she died (Métraux, 1980); she also edited *Margaret Mead: Some Personal Views* (1979). Lapsley (1999) has highlighted that while less is known about this relationship, Mead shared more of her life with Métraux than anyone else, and it was known by those close to them that they were lovers (though this may not have been the case throughout the entire time they knew one another). Likewise, Banner (2010: 431) states that it was following an affair with Gregory Bateson that Métraux became ‘Margaret’s partner for the rest of her life’.

There is therefore the need to situate this exchange within the confines of what Mead and Métraux were likely to feel able to say within this interview. It is highly unlikely Sanctuary would have had any knowledge of Mead’s previous lesbian relationships, and it is also unlikely he would have known the two women he was interviewing were in a lesbian relationship at the time. Mead’s relationships with other women were revealed publicly only posthumously, and there is no evidence Sanctuary knew Mead or Métraux personally or prior to the interview. Mead’s own response about how it can be ‘extraordinarily hard to tell’ opens up queer potential and draws in a wider network of other women she knows in similar situations. Indeed, the characteristics of the ‘friends’ she introduces echoes some aspects of her own relationship with Ruth Benedict (14 years her senior) and later Métraux (13 years her junior). It could also be referring to Benedict, who later understood herself as a lesbian and had relationships with other women including Natalie Raymond (Banner, 2003) and Ruth Valentine. Mead may also have understood Lowenfeld and Anderson’s relationship to be potentially similar to a long-term domestic relationship she had with Marie Eichelberger, which was for the most part neither sexual nor romantic but nonetheless breached heteronormative friendship boundaries (see Banner, 2003). Of course, both Mead and Métraux also had relationships with men, much as Lowenfeld likely had with Collingwood. But these relationships with men do not of course negate their queerness, or the significance of their lesbian relationships. Indeed, Mead hypothesised that almost everyone is bisexual, or of ‘mixed type’, as she called it (Banner, 2003). The danger lies in a heteronormative narrative that emphasises solely these relationships at the expense of the queer ones this network of women were having.

## Conclusion

In considering the relationships between these women, it has been possible not only to reveal the type of uneven working relationship between Lowenfeld and Mead, but also to further reveal the queer intricacies of their lives. Mead and Métraux’s responses to Sanctuary’s questions in these interviews were constrained by social norms, historical context, terminology, and a lack of intersubjectivity. Yet, by analysing these transcripts now from a queer feminist perspective, it is possible to reveal more and open up queer potential. Crucially, this also demonstrates what a lesbian feminist historical approach uniquely provides. It is clear that the relationship between Lowenfeld and Anderson was in many ways an impossible love, a relationship described in ways that shy away from, rather than attend to, the intimacy they clearly shared. Likewise, there appears to have been some kind of shared understanding between Lowenfeld and Mead – a recognition of them both being women who have not gone the ‘common way’, to use a term used by Lowenfeld (Hubbard, 2020: 70). Lowenfeld is described as being captivating in various ways, and I argue that via this ‘capture’ a queer kinship was formed between Lowenfeld and Mead and others.

In recognising the queer kinships between Mead, Lowenfeld, Anderson, and Métraux, I also reveal the reflexive and emotional queer kinship that extends between researcher and subject. It is this queer kinship across historical time that allows for more nuanced and emotionally sensitive history. I deliberately experience this queer kinship as a highly valued aspect of intersubjectivity, which has allowed me to interpret via an affirmative queer lens in ways that were not possible at the point when these interviews took place. Such reflective practice and transparency around historical work is relatively rare, yet I feel there is significant value in applying reflexivity to such methods. Lepore (2001), for example, talks about the intimacy of conducting biography. Mead similarly discussed having a sort of substitute intimacy with Lowenfeld through the discussion of ‘cases’. Here, I feel there has been a similar kind of intimacy, one aligned to queer kinship found and established in the archive.

In keeping with Love (2009: 30), I too feel ‘attuned to the queer historical experience of failed or impossible love and have tried to make this feeling the basis for my own approach to the past’. Lowenfeld’s influence therefore lives on, perhaps not so much in psychology, but certainly in the archive, awaiting ‘the touch of the queer historian’, to paraphrase Love (ibid.). I also wish to identify the moment in the interview when Sanctuary asked, ‘Do you think the relationship with Ville was a homosexual one?’ as a moment of queer touch. In this moment, Mead and Métraux were confronted by their own queerness, as well as that of Lowenfeld and Anderson. Together, in the archive, over 40 years later, there was a queer kinship between us all as I too reached back in time and was captured by Lowenfeld once more.

## Notes

Many thanks to the Wellcome Trust for funding for the project ‘Dear Margaret: The Interconnected Lives and Work of Queer Women in 20th Century British Social Science’ (grant no. 212711/Z/18/Z), which also provides open access for this article. I also want to acknowledge the talk I gave at the Wellcome Library in 2020, which further sparked this project through conversations with Rhodri Hayward, Lesley Hall, and the members of the Margaret Lowenfeld Trust. Many thanks also to David Griffiths and Clare Butler for reading previous drafts, and to Mo Moulton for talking the article through, as well as the reviewers, who provided very useful comments and encouragement. The writing of this article was greatly aided by the Psychology of Women and Equality 2022 writing retreat – thanks for the space and the swim.

1. Wellcome Library, London, PP/PRE/J.1/25.

2. Mead has of course considered kinship in her own anthropological work, and the concept has also been used to consider her own relationships with women (see Lapsley, 1999).

3. Margaret Mead and Rhoda Métraux’s extensive collection is archived at the Library of Congress in Washington, DC.

4. The Lowenfeld collection is located at the Wellcome Library, London, and her collection of objects for the World Technique is housed at the Science Museum. Her legacy is also managed by the Margaret Lowenfeld Trust, and her library of books and some tests is housed at the Centre for Family Research at the University of Cambridge.

5. Wellcome Library, PP/PRE/J.1/25.

6. Wellcome Library, PP/PRE/J.1/25, specifically Tape 32, which is the recording of the interview with Margaret Mead and Rhoda Métraux by Gerald Sanctuary on 17/18 January 1977. I have used the Lowenfeld collection and the Helena Wright collection as the contextual basis for this article, as I am familiar with the contents of both through years of research in this area.

7. See Banner (2003) for a comprehensive account of how Mead considered sexuality, masculinity and femininity, and gender. This includes consideration of how Mead understood her own sexuality and that of those around her, including Ruth Benedict. While we might understand Mead as bisexual today, she more often used the term ‘mixed type’ and to some extent denied Benedict’s later lesbian identification.

8. During the mid 20th century, the psy-disciplines and medicine, the area Lowenfeld had been educated and was working in, had taken ownership of homosexuality as a ‘problem’ leading to pathologisation and supposed ‘treatments’. Stigma was incredibly high and, as articulated elsewhere, the history of psychology in relation to LGBTQI people was, and still is, complex (see Dickinson, 2015; Hegarty, 2017; Hubbard and Griffiths, 2019; Spandler and Carr, 2020).

 9. For discussion and reflection on these issues with particular reference to Lowenfeld and others, see Hubbard (2017, 2018, 2020).

10. This property is now the Irena and Mieczysław Mazaraki Museum, showcasing exhibitions on the history and culture of the region.

11. The British Society for the Study of Sex Psychology was founded in 1913 on the basis of the writings of Edward Carpenter and Havelock Ellis and was very open-minded and progressive. In the details that are available about Lowenfeld’s involvement, it is clear she did not wish to be associated with Norman Haire (who was an Australian sexologist). Lowenfeld’s distancing was potentially related to a falling-out he had with Stella Browne (a feminist birth control campaigner), and she also gave a talk in 1933 (see Hall, 1995).

12. In Lowenfeld’s will, Anderson was allocated all assets in one fund and also given technical and professional equipment, plus Lowenfeld’s motorcar. Both she and Mead were identified as key people to administer her wishes in the will regarding ICP funding.

13. The video can be found at Wellcome Library, PP/LOW/Z/4.

14. Wellcome Library, PP/PRE/J.1/25, Tape 32 (see n. 6). All subsequent quotations are from this source unless otherwise specified.

15. For example, Collingwood drew sketches of the children’s play and copies of the worlds made for the Lowenfeld World Technique. He also gave a lecture series at the ICP and ran training seminars for the staff there (Hayward, 2020).
